# Comparative Genomic Data Provide New Insight on the Evolution of Pathogenicity in *Sporothrix* Species

**DOI:** 10.3389/fmicb.2020.565439

**Published:** 2020-09-30

**Authors:** Mengya Huang, Ziying Ma, Xun Zhou

**Affiliations:** ^1^Department of Dermatology and Cosmetology, Chongqing Traditional Chinese Medicine Hospital, Chongqing, China; ^2^College of Traditional Chinese Medicine, Chongqing Medical University, Chongqing, China; ^3^State Key Laboratory of Mycology, Institute of Microbiology, Chinese Academy of Sciences, Beijin, China

**Keywords:** *Sporothrix*, comparative genomics, enzyme, gene clusters, phylogeny, fungal evolution

## Abstract

*Sporothrix* species are commonly isolated from environmental and clinical samples. As common causes of zoonotic mycosis, *Sporothrix* species may result in localized or disseminated infections, posing considerable threat to animal and human health. However, the pathogenic profiles of different *Sporothrix* species varied, in virulence, geographic location and host ranges, which have yet to be explored. Analysing the genomes of *Sporothrix* species are useful for understanding their pathogenicity. In this study, we analyzed the whole genome of 12 *Sporothrix* species and six *S. globosa* isolates from different clinical samples in China. By combining comparative analyses with Kyoto Encyclopedia of Genes and Genomes (KEGG), Carbohydrate-Active enZymes (CAZy), antiSMASH, Pfam, and PHI annotations, *Sporothrix* species showed exuberant primary and secondary metabolism processes. The genome sizes of four main clinical species, i.e., *S. brasiliensis*, *S. schenckii*, *S. globosa*, and *S. luriei* were significantly smaller than other environmental and clinical *Sporothrix* species. The contracted genes included mostly CAZymes and peptidases genes that were usually associated with the decay of plants, as well as the genes that were associated with the loss of pathogenicity and the reduced virulence. Our results could, to some extent, explain a habitat shift of *Sporothrix* species from a saprobic life in plant materials to a pathogenic life in mammals and the increased pathogenicity during the evolution. Gene clusters of melanin and clavaric acid were identified in this study, which improved our understanding on their pathogenicity and possible antitumor effects. Moreover, our analyses revealed no significant genomic variations among different clinical isolates of *S. globosa* from different regions in China.

## Introduction

*Sporothrix* species distribute all over the world, particularly in tropical and subtropical areas. *Sporothrix* includes about 51 species, commonly isolated from environmental and clinical samples (de Beer et al., 2016). Some *Sporothrix* species are associated with plants, decaying wood and organics in soil as environmental saprobes, while some are agents of Sporotrichosis on animals and humans (Rodrigues et al., 2016).

Sporothricosis is common in horticulturists, farmers and miners exposed to plants, soil or organic matter contaminated with *Sporothrix* pathogens, and is also seen in animals such as cats and dogs (Barros et al., 2011). The most common clinical lesion types are lymphocutaneous and fixed localized lesions (Verma et al., 2012). In addition, disseminated sporotrichosis might develop in immunocompromised patients (White et al., 2019).

Variations exist between different *Sporothrix* species including virulence, morbidity, host preference and drug resistance (Arrillaga-Moncrieff et al., 2009; Zhang et al., 2015). The four main species well known as pathogenic to humans and animals are *S. brasiliensis*, *S. schenckii*, *S. globosa*, and *S. luriei* (Zhang et al., 2015). Among them, *S. brasiliensis* is the most virulent species, followed by *S. schenckii*, *S. globosa*, and *S. luriei* (Arrillaga-Moncrieff et al., 2009; Zhang et al., 2015). Sporotrichosis caused by *S. schenckii* is prevalent globally (Chakrabarti et al., 2015), While *S. globosa* prevails in Asia, Europe and the Americas (Camacho et al., 2015). Recent evidences suggested that *S. globosa* is the major Chinese pathogenic species, with broad geographic distribution (Moussa et al., 2017; Nesseler et al., 2019; Song et al., 2013). *S. brasiliensis* is known to be always transmitted by cats and has outbroken in Brazil (Mora-Montes et al., 2015). *S. luriei* is a relatively rare pathogen compared with the other three species (Padhye et al., 1992; Marimon et al., 2008; Oliveira et al., 2011). Infections were also occasional reported from other species, i.e., *S. mexicana* (Dias et al., 2011) and *S. pallida* (Rodrigues et al., 2013). While *S. humicola* was known to be pathogenic to animals, as well as saprobic in the environment (Nesseler et al., 2019).

As far, previous multi-locus phylogenetic analyses have inferred that all clinical species have evolved from environmental species (Song et al., 2013; Zhou et al., 2014; Zhang et al., 2015). Several studies reported the whole-genome of few pathogenic species (D’Alessandro et al., 2016; de Beer et al., 2016; Huang et al., 2016; Shang et al., 2016), but the differences in genomes between environmental strains and clinical strains were never compared, posing difficulties in explaining the relationships between phenotypes and genotypes in the evolution of *Sporothrix*. To further understand the variations in the pathogenicities of *Sporothrix*, we analyzed and annotated the genomes and secondary metabolite biosynthesis of the four main clinical species, together with other rare pathogens and environmental species. Six clinical strains of *S. globosa* isolated from different regions in China were also compared and analyzed to understand their geographic variations.

## Materials and Methods

### Fungal Strains and DNA Extraction

Six *Sporothrix* strains isolated from the skin lesions of patients in China and 10 ex-type strains obtained from the CBS-KNAW Fungal Biodiversity Centre, including *S. globosa*, *S. schenckii luriei*, *S. mexicana*, *S. pallida*, *S. brunneoviolacea*, *S. dimorphospora*, *S. humicola*, *S. inflata*, *S. lignivora*, and *S. variecibatus* were included in the study (Table 1). Isolates were inoculated on 2% (0.02 g/ml) potato dextrose agar (PDA) and incubated for 7 days at 25°C prior to harvesting 100–200 milligrams of fungal mycelia. Genomic DNA was subsequently extracted using the cetyltrimethyl ammonium bromide (CTAB) method (Liu et al., 2013).

**TABLE 1:** Summary of isolates used in this study.

Strain	Name	Source	Origin
LC 2460	*S. globosa*	Clinical strain	Beijing, China
LC 2454	*S. globosa*	Clinical strain	Beijing, China
LC 2445	*S. globosa*	Clinical strain	Chongqing, China
LC 2433	*S. globosa*	Clinical strain	Chongqing, China
LC 2405	*S. globosa*	Clinical strain	Jilin, China
LC 2404	*S. globosa*	Clinical strain	Jilin, China
CBS 120340	*S. globosa*	Clinical strain	Spain
CBS 937.72	*S. luriei*	Clinical strain	South Africa
CBS 131.56	*S. pallida*	Stemonitis fusca	Japan
CBS 120341	*S. mexixcana*	Soil	Mexico
CBS 118129	*S. humicola*	Soil	South Africa
CBS 553.74	*S. dimorphospora*	Soil	Canada
CBS 239.68	*S. inflata*	Wheatfield soil	Germany
CBS 121961	*S. variecibatus*	Protea infructescences	South Africa
CBS 124561	*S. brunneoviolacea*	Soil	Spain
CBS 119148	*S. lignivora*	Eucalyptus wood pole	South Africa

### Genome Sequencing and Assembly

The whole genomes of *Sporothrix* strains were sequenced using next generation Illumina Hiseq 2000 technology. Analysis based on K-mer using Jellyfish^1^ was undertaken to estimate the error rate of sequencing and the sizes of the genomes including heterozygosity and duplication. The sequence reads of *Sporothrix* genomes were assembled into contigs and scaffolds using SPAdes^2^. The effect of the contigs and scaffolds assembled was assessed with the default command of quast. Only scaffolds longer than 200 bp were considered for further analyses.

### *Ab initio* Gene Prediction and Annotation

The genomes of *S. schenckii* (AWEQ00000000.1, strain ATCC 58251) (Lombard et al., 2014) and *S. brasiliensis* (AWTV00000000.1, strain 5110) (de Beer et al., 2016) were downloaded from the GenBank, and were analyzed together with our strains. Gene predictions were performed using GeneMark-ES (Borodovsky and Lomsadze, 2011) in unsupervised *ab initio* mode. Automatic annotations were performed using Kyoto Encyclopedia of Genes and Genomes (KEGG)^3^, Pfam (downloaded in June 2019) and PHI (version 4.8) databases (Han et al., 2016; Song et al., 2020) to analyze both homology and specific proteins. All results of Pfam and PHI databases were obtained using locally compiled databases. KEGG Pathways were inspected manually to analyze the metabolic pathways. The assembled scaffolds of the *Sporothrix* genomes were supplied to antiSMASH^4^ to evaluate the secondary metabolites (Blin et al., 2019). Gene clusters involved in pathogenicity, virulence and other areas of interest were further examined according to the domain annotations. BLASTp was adopted to search against the Gene Ontology (GO) database (by August 2019). Annotation of carbohydrate-active enzymes was performed with the translated protein sequences using the Carbohydrate-Active enZymes (CAZy) database^5^ (Lombard et al., 2014).

### Statistical Analysis

Data were analyzed using STATA version 14.2 (Stata Corporation, College Station, United States). Normal distributed data were analyzed using *t*-test, while non-normal distributed data were analyzed using the Wilcoxon sign rank test. A *p* < 0.05 was considered as statistically significant.

## Results

### Characteristics of the *Sporothrix* Genomes

The genome assembly of 16 *Sporothrix* strains ranged from approximately 33–44 Mb and included 138–5460 scaffolds with N50 length ranging from 1.8 to 10.7 Mb. The k-mer frequency distribution showed a unique peak suggesting that the sequenced fungal genomes did not exhibit significant heterozygosity. The number of predicted proteins ranged from 9010 to 13344. Genomes assemblies of the 16 *Sporothrix* strains were deposited in the GenBank. In this study, genome statistics including accession numbers, genome size, predicted proteins, assembled contigs and scaffolds were shown in Table 2. The genome size was largest in *S. lignivora*, followed by *S. mexixcana*, *S. humicola*, *S. pallida*, *S. inflata*, *S. dimorphospora*, *S. variecibatus*, *S. brunneoviolacea*, *S. luriei*, *S. globosa*, *S. brasiliensis*, and *S. schenckii*. There was no significant difference in genomes of Chinese *S. globosa* isolates, as well as with the ex-type strain.

**TABLE 2:** Genome statistics of *Sporothrix*.

Strain	Name	GenBank accession number	Genome size (Mb)	G + C content (%)	N50 length (Mb)	Predicted proteins	Assembled contigs	Assembled scaffolds	References
5110	*S. brasiliensis*	AWTV00000000.1	33.21	54.7	3.8	9091	601	–	de Beer et al., 2016
ATCC 58251	*S. schenckii*	AWEQ00000000.1	32.23	55.1	3.3	8674	125	29	Lombard et al., 2014
CBS 120340	*S. globosa*	WOEH00000000	33.29	54.51	2.9	9009	779	312	–
LC 2460	*S. globosa*	WNYM00000000	33.36	54.47	3.6	9036	837	276	–
LC 2454	*S. globosa*	WNYL00000000	33.36	54.46	3.1	9039	761	277	–
LC 2445	*S. globosa*	WNYK00000000	33.35	54.47	3	9128	817	308	–
LC 2433	*S. globosa*	WNYJ00000000	33.39	54.51	2.7	9098	819	371	–
LC 2405	*S. globosa*	WMHP00000000	33.44	54.42	2.3	9218	753	414	–
LC 2404	*S. globosa*	WNYB00000000	33.43	54.47	5	9010	2016	241	–
CBS 937.72	*S. luriei*	WNLO00000000	34.24	55.75	1.8	10,105	16,679	2213	–
CBS 120341	*S. mexixcana*	WNYC00000000	43.74	52.79	4.5	11,997	40,495	5460	–
CBS 131.56	*S. pallida*	WNYG00000000	40.23	52.76	4.8	10,749	1451	342	–
CBS 118129	*S. humicola*	WNYE00000000	40.74	52.21	3.6	10,656	2120	447	–
CBS 553.74	*S. dimorphospora*	WOUA00000000	39.13	55.17	5.6	10,706	10,821	598	–
CBS 239.68	*S. inflata*	WNYF00000000	39.53	55.47	3	11,008	1787	347	–
CBS 121961	*S. variecibatus*	WNYH00000000	38.87	52.94	10.7	10,411	738	138	–
CBS 124561	*S. brunneoviolacea*	WNYD00000000	37.75	56.41	1.9	11,160	8508	825	–
CBS 119148	*S. lignivora*	WNYI00000000	43.98	51.2	8.3	13,344	12,426	1438	–

### Gene Expansion and Contraction in the *Sporothrix* Species

Evolutions of *Sporothrix* in gene families were inferred based on domain expansions or contractions assigned by the antiSMASH, CAZy, KEGG, Pfam, and PHI databases.

**FIGURE 1:**
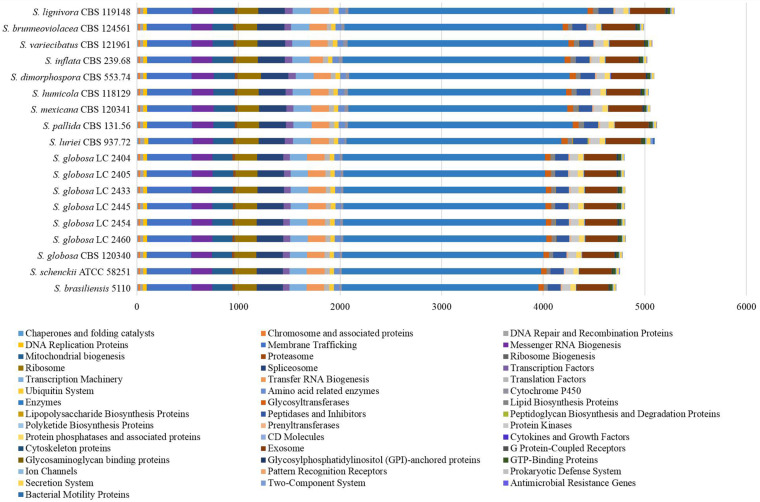
Protein annotation of KEGG database in *Sporothrix.* All proteins annotated with known function were classified into different categories according to the KEGG database. The core metabolic functions comprised enzymes, exosome, membrane trafficking, messenger RNA biogenesis, mitochondrial biogenesis, ribosome, and spliceosome.

**FIGURE 2:**
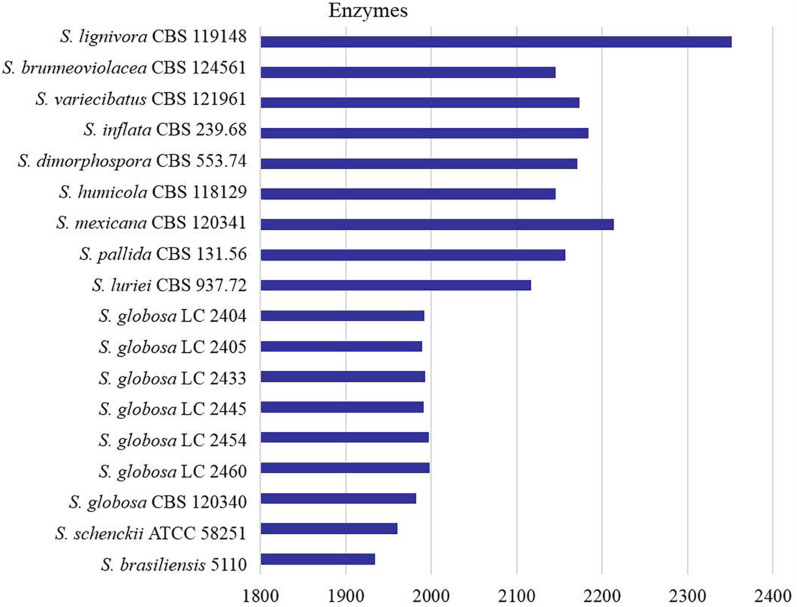
Counts of the enzymes encoded by genes in *Sporothrix.* Enzyme genes of four main clinical species (*S. brasiliensis*, *S. schenckii*, *S. globosa* and *S. luriei*) were less than most of environmental and other clinical species.

All proteins annotated with known function were classified into different categories according to the KEGG database (Figure 1). We found that core metabolic functions comprised enzymes, exosome, membrane trafficking, messenger RNA biogenesis, mitochondrial biogenesis, ribosome, and spliceosome. Enzymes metabolism was significantly predominant in contrast to other classifications. The enzyme genes of four main clinical species (*S. brasiliensis*, *S. schenckii*, *S. globosa*, and *S. luriei*) were less than most of environmental and other clinical species (Figure 2).

**FIGURE 3:**
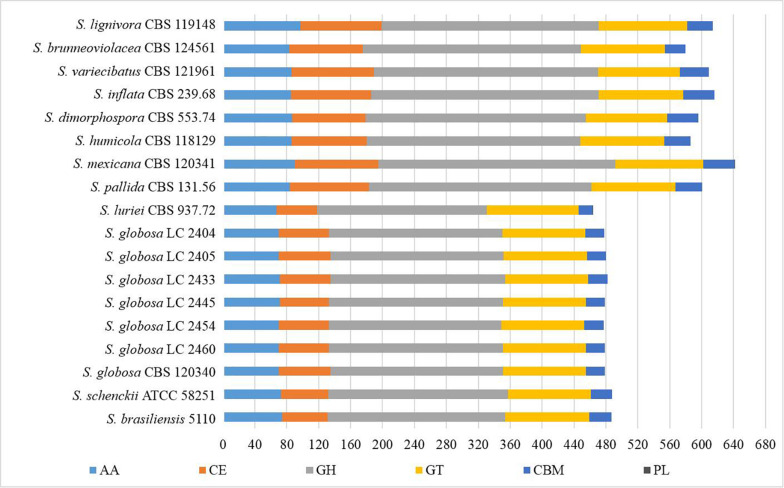
Gene contraction of carbohydrate-active enzymes (CAZymes) in the genus *Sporothrix.* Genes of *Sporothrix* identified through CAZy database were classified into 6 categories. CAZymes genes of the four main clinical species (*S. brasiliensis*, *S. schenckii*, *S. globosa*, and *S. luriei*) were significantly less than those of the other *Sporothrix* species, especially the genes belonging to the GH and CE groups.

**FIGURE 4:**
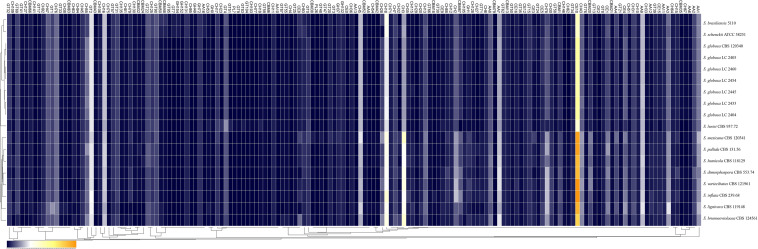
Comparison of the CAZymes identified in the genomes of the genus *Sporothrix.* CAZy-coding genes falling into 141 families and CBM-coding genes in 19 families were identified in the genomes of the genus *Sporothrix*. There was a higher abundance of PPD enzyme genes, especially GH1, GH2, GH3, GH28, GH43, GH78, and CE1 in two clinical species (*S. mexicana* and *S. pallida*) and environmental species. A remarkable gene contraction was observed in the four main clinical species (*S. brasiliensis*, *S. schenckii*, *S. globosa*, and *S. luriei*) including AA3, AA7, CBM20, and CE10 families. Genes of CBM48 and CBM50 expanded in *S. brasiliensis* and *S. schenckii.*

We explored the *Sporothrix* genomes for genes encoding carbohydrate-active enzymes and carbohydrate-binding modules. CAZy-coding genes falling into 141 families and CBM-coding genes in 19 families were identified. These genes were categorized into the auxiliary activities (AA), glycoside hydrolases (GH), glycosyltransferases (GT), carbohydrate-binding modules (CBM), carbohydrate esterases (CE), and polysaccharide lyases (PL) families. We observed that the highest proportion of gene sequences were assigned to the GH family, while there were a lack of polysaccharide lyase genes in almost all *Sporothrix* strains. A few gene sequences related to subfamilies PL1 and PL27 were detected only in *S. luriei* and *S. mexicana*. Further analysis was carried out to determine the gene distribution in the respective classifications. The Wilcoxon rank-sum test based on the gene counts in these families showed that there were significant differences (*p* < 0.05) in the distribution of the enzyme genes between the four main clinical species and other *Sporothrix* species. The heatmap and chart based on family classification suggested that the enzyme genes of the four main clinical species were significantly less than those of the other *Sporothrix* species, especially the genes belonging to the GH and CE groups (Figures 3, 4).

**FIGURE 5:**
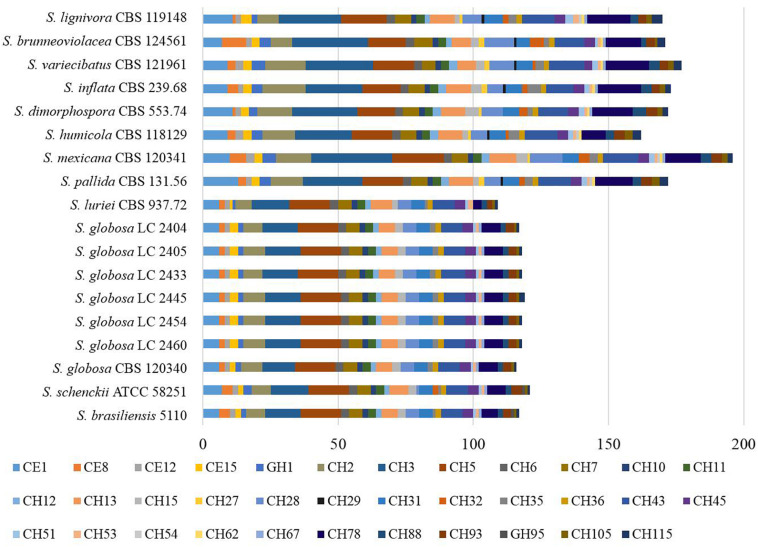
Counts of the putative genes involved in plant polysaccharide degradation in the genus *Sporothrix.* The major plant polysaccharide degradation (PPD) enzyme genes in the four main clinical species (*S. brasiliensis*, *S. schenckii*, *S. globosa*, and *S. luriei*) were less than those of the environmental and other clinical species.

Most CAZy enzyme genes were predicted using comprehensive annotation and comparison analysis in the CAZy database. The major plant polysaccharide degradation (PPD) enzyme genes in the four main clinical species were less than those of the environmental and other clinical species in further analyses (Figure 5). There was a higher abundance of PPD enzyme families, especially GH1, GH2, GH3, GH28, GH43, GH78, and CE1 in two clinical species (*S. mexicana* and *S. pallida*) and environmental species (Figures 4, 5). In addition, a remarkable contraction was observed in the four main clinical species including AA3 and AA7 families supporting the lignocellulose degradation, CBM20 carbohydrate-binding modules enhancing the activity of granular starch-binding function, and CE10 families assisting great capacity to digest pectin (Figures 4, 5). Though most CAZymes genes were in contraction, the comparative analysis revealed genes of CBM48 and CBM50 expanded in the two most virulent species, *S. brasiliensis* and *S. schenckii* (Figures 4, 5).

**FIGURE 6:**
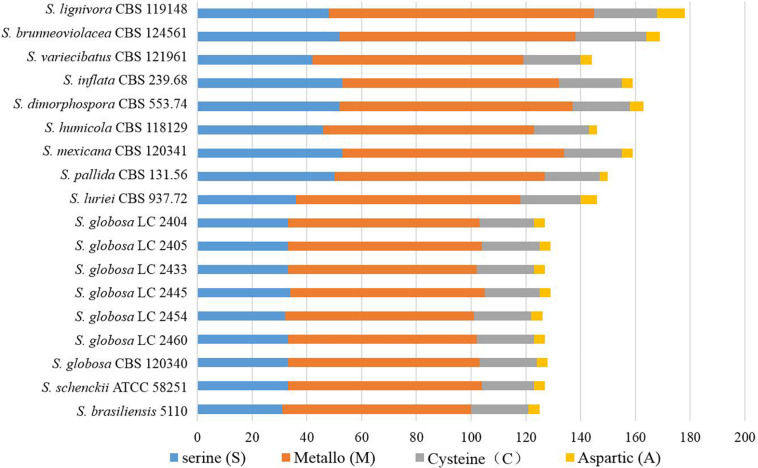
Gene contraction of peptidases in the genus *Sporothrix.* Protease-coding genes of *Sporothrix* identified through Pfam database were classified into 4 categories. The largest category of predicted proteases in the *Sporothrix* genome was Metallo (M), followed by Serine (S), Cysteine (C), and Aspartic (A) proteases.

**FIGURE 7:**
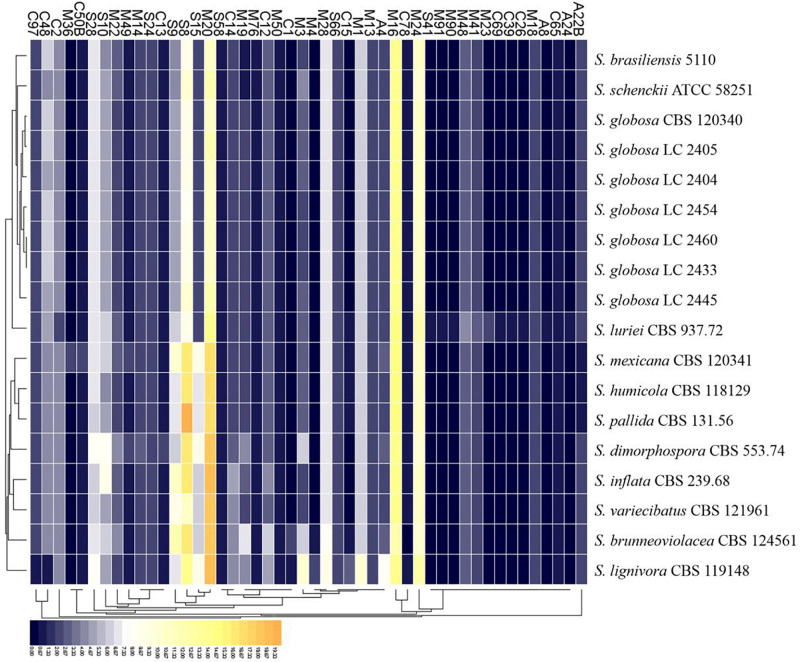
Heatmap of the peptidases identified in the genomes of the genus *Sporothrix.* Protease-coding genes of *Sporothrix* identified through Pfam database were classified into 48 subfamilies. The four main clinical species (*S. brasiliensis*, *S. schenckii*, *S. globosa*, and *S. luriei*) had less M20, S8, S9, and S15 subfamily genes than other species.

There were 125–178 protease-coding genes identified through Pfam database, which were classified into 4 categories consisting of 48 subfamilies (Figures 6, 7). In general, the largest category of predicted proteases in the *Sporothrix* genome was Metallo (M), followed by Serine (S), Cysteine (C), and Aspartic (A) proteases (Figure 6). No enrichment of peptidase genes in the clinical species was observed. The four main clinical species had less M20, S8, S9, and S15 subfamily genes than other species (Figure 7).

**FIGURE 8:**
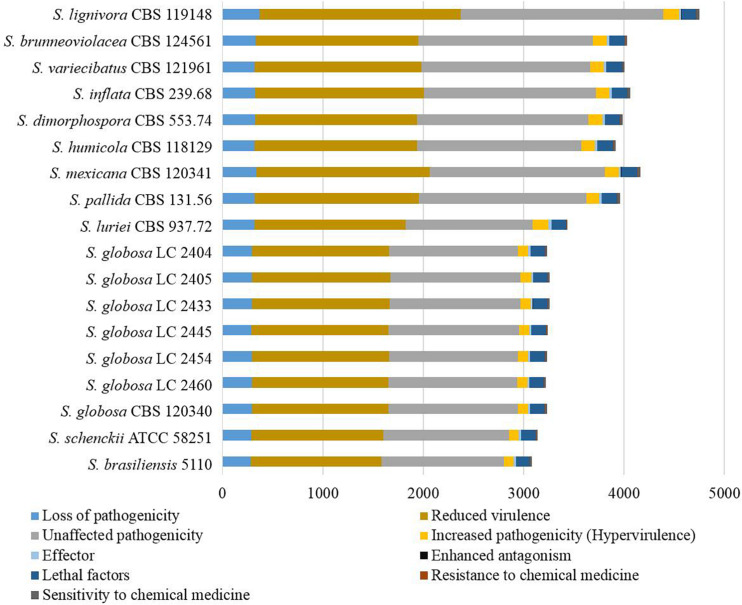
Genes associated with virulence in the genus *Sporothrix.* From the virulence gene prediction, genes of five classifications, i.e., loss of pathogenicity, reduced virulence, unaffected pathogenicity, increased pathogenicity, and lethal factors in four main clinical species (*S. brasiliensis*, *S. schenckii*, *S. globosa*, and *S. luriei*) were less than those in the environmental and other clinical species, and the reduced number of genes associated with the loss of pathogenicity, reduced virulence and unaffected pathogenicity was far more than that associated with the increased pathogenicity and lethal factors.

**FIGURE 9:**
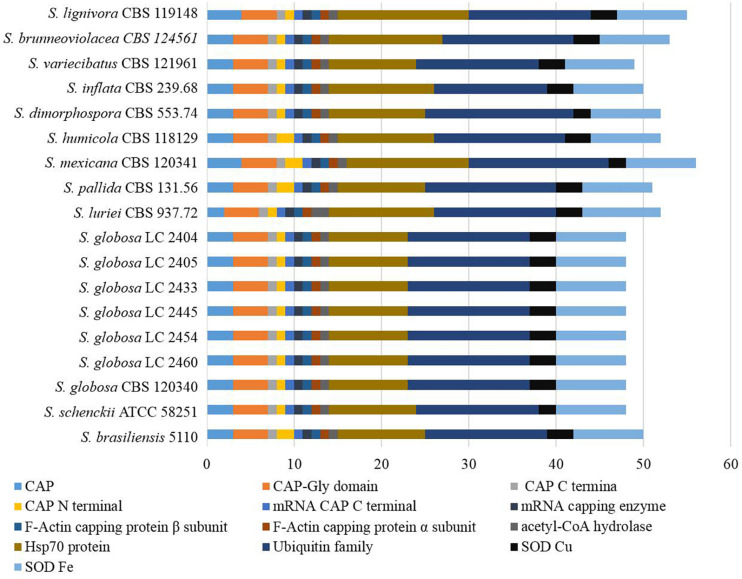
Virulence genes annotated with Pfam in the genus *Sporothrix.* Virulence gene families assigned by Pfam were summarized into cyclase-associated protein (CAP), Coenzyme A (CoA), heat shock protein 70 (Hsp70), ubiquitin family, and superoxide dismutases (SOD). Significant difference in the five classifications of virulence genes was not observed in different *Sporothrix* species.

Genes associated with virulence of 16 *Sporothrix* strains ranged from 3083 to 4750. The highest number was found in *S. lignivora* (CBS 119148), and the lowest in *S. brasiliensis* (5110) (Figure 8). Genes associated with the reduced virulence, and unaffected pathogenicity were consistently present with higher copy numbers in all strains. From the virulence gene prediction, genes of five classifications, i.e., loss of pathogenicity, reduced virulence, unaffected pathogenicity, increased pathogenicity, and lethal factors in four main clinical species were less than those in the environmental and other clinical species (*p* = 0.000000040, Wilcoxon sign rank test), and the reduced number of genes associated with the loss of pathogenicity, reduced virulence and unaffected pathogenicity was far more than that associated with the increased pathogenicity and lethal factors. Virulence gene families assigned by Pfam in this study were summarized into the following five classifications, i.e., cyclase-associated protein (CAP), Coenzyme A (CoA), heat shock protein 70 (Hsp70), ubiquitin family, and superoxide dismutases (SOD). However, significant difference in the five classifications of virulence genes was not observed in the different *Sporothrix* species (Figure 9). Genes associated with effector, enhanced antagonism, resistance to chemical medicine and sensitivity to chemical medicine present lower copy numbers, with no significant difference among different *Sporothrix* species (Figure 8).

### Bioactive Secondary Metabolites

**FIGURE 10:**
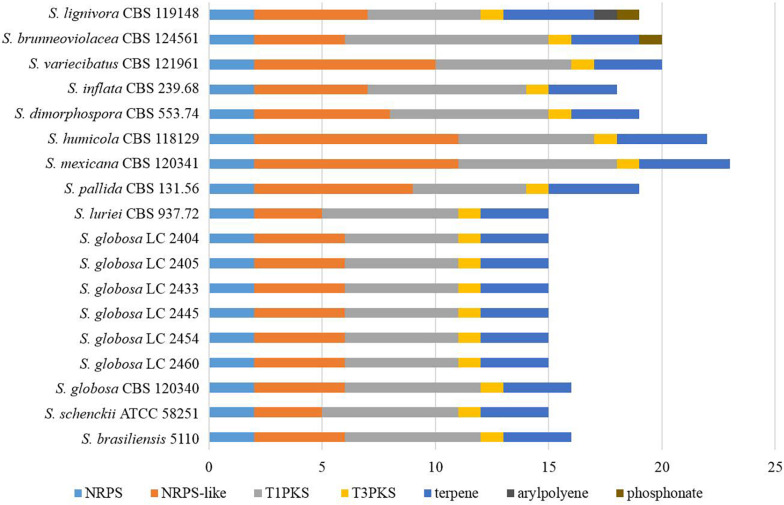
The putative secondary biosynthetic gene clusters identified in the genomes of *Sporothrix* species. Secondary biosynthetic gene clusters of *Sporothrix* were identified though antiSMASH platform. These putative gene clusters belonged to *tIpks*, *nrps*, terpene, *tIIIpks*, indole, arylpolyene, and phosphonate. Gene clusters of *tIpks* and *nrps* were less in the four main clinical species (*S. brasiliensis*, *S. schenckii*, *S. globosa*, and *S. luriei*) than those of the environmental and other clinical species.

Fifteen to 23 putative secondary biosynthetic gene clusters were identified though the antiSMASH platform. There were 15–16 gene clusters in the four main clinical species, while 17–23 gene clusters in other species. Many of these putative gene clusters belonged to type I *pks* (*tIpks*), non-ribosomal peptide synthase (*nrps*) gene clusters, and terpene, while the rest were type III *pks* (*tIIIpks*), indole, aryl polyene, or phosphonate. The gene clusters of *tIpks* and *nrps* were less in the four main clinical species. The number of *tIIIpks* genes was the same in all *Sporothrix* species (Figure 10).

All sequenced *Sporothrix* strains had *tIpks* gene clusters encoding melanin, and terpene gene clusters encoding clavaric acid. Based on the BLASTp and domain analyses, we identified 58.3% strains with emericellin genes in the *nrps* gene clusters and 41.7% strains with non-acetylated open-chain sophorolipid genes in the *tIpks* gene clusters. We detected that 75% strains have *tIpks* genes with 33.3% sequence identity with fujikurins. In addition, several predicted gene clusters of two clinical species (*S. mexicana* and *S. pallida*) and environmental species might encode HC-toxin, phenalamide, lydicamycin, TAN-1612, trypacidin, aspercryptins, usnic acid, botryenalol, melithiazol, leporin, dehydrocurvularin, sorbicillin, elsinochrome A, equisetin, clapurines, and natamycin.

The ex-type strain of *S. globosa* was compared with the clinical strains from three regions in China (Beijing, Chongqing, Jilin). Though the incidence of sporotrichosis and the growth rate of colony in three regions were different, there was no significant difference in CAZyme-coding genes (Figures 3–5), protease-coding genes (Figures 6, 7), virulence related genes (Figures 8, 9) and putative secondary biosynthetic gene clusters (Figure 10) between the ex-type strain and Chinese clinical strains.

## Discussion

*Sporothrix* species infect humans and animals, and are widely distributed in the environments. The recent outbreaks in Australia, Brazil, China, India and South Africa have raised serious concerns on public health. *Sporothrix* is one of the major causes of deep fungal infection, with increasing resistance to antifungal medicines (Li J. et al., 2019). *Sporothrix* species have shown considerable variations in virulence, host preference and drug resistance, and the study on the relationships between phenotypes and genotypes of *Sporothrix* is crucial for understanding their pathogenesis.

Differences in pathogenic phenotypes of *Sporothrix* species could be correlated to the expansion or contraction in specific gene families. Previous multi-locus phylogenetic studies could not explain these phenotypic variations. Next-generation sequencing enables a comprehensive profiling of genetic data which enables a better interpretation and correlation of the phenotypic characters with their genetic bases. Previous studies speculated that the clinical species evolved from environmental species (Zhou et al., 2014), and the four main clinical species (*S. brasiliensis*, *S. schenckii*, *S. globosa*, and *S. luriei*) proved to show high degrees of endemicity (Zhang et al., 2015). In this study, the four main clinical species have smaller genome sizes and the number of predicted proteins are also obviously less, as compared to the environmental species (*S. brunneoviolacea, S. dimorphospora, S. humicola, S. inflata, S. lignivora, and S. variecibatus*) and other clinical pathogens (*S. mexicana* and *S. pallida*). Therefore, we infer that genes lost in the evolution of *Sporothrix*. Gene loss is a pervasive route of genetic contraction. On the other hand, gene gain is usually a main driver of genetic expansion (Zhi et al., 2017). Gene loss or gain as a source of functional variations plays a prominent role in microbial evolutions (Bush et al., 2017). We consider that the gene contraction, i.e., the abandonment of genes is more important, as compared to the gene expansion in the evolution of *Sporothrix*.

The number of enzyme genes significantly differ in *Sporothrix* species. CAZymes are important in the degradation of carbohydrate in the hosts. PPD enzymes of CAZymes provide carbon sources by degrading plant polysaccharides in cytoderm (Courtade and Aachmann, 2019; Pan et al., 2019). Poverty in CAZymes genes related to degrading cellulose, xyloglucan, galactomannan, and pectin, is not surprising in the four main clinical *Sporothrix* species, as humans and animals lack cytoderm. The obvious contraction of these enzyme genes in the four main clinical species are suggestive of reduced plant invasive capabilities. On the other hand, a clear expansion of enzyme genes in CBM48 and CBM50 domains is observed in two most virulent pathogens *S. brasiliensis* and *S. schenckii*. CBM48, similar to CBM20, is mostly involved in starch biosynthesis (Wilkens et al., 2018). CBM50, also known as LysM domains, plays an important role in inhibiting host defenses (Volk et al., 2019). Peptidases provide alternative carbon sources for glucose starving cells through degrading proteins (Handtke et al., 2018), and promote various progresses, such as cell growth and differentiation, apoptosis, cell cycle, and signaling (Ng et al., 2009). Mutant in peptidase gene has been known as an important drive for fungal adaptation to hosts and environment (Soberanes-Gutierrez et al., 2015; Cervantes-Montelongo and Ruiz-Herrera, 2019). Interestingly, none of the genes related to Asparagine, Glutamate, and Threonine were previously predicted. Moreover, we have not observed the enrichment of peptidase genes in clinical *Sporothrix*, specifically M35 and M36 subfamilies, which usually expand in dimorphic fungal pathogens as an adaptation to mammalian hosts (Sharpton et al., 2009). This result is consistent with that of Teixeira (Teixeira et al., 2014). In contrast, we observed less M20, S8, S9, and S15 subfamily genes in these strains. A previous study showed that clinical fungi tended to have less prolyl oligopeptidases (S9), and subtilisins (S8) that might be associated with a saprotrophic lifestyle (Muszewska et al., 2017). The occurrence of genes contraction in CAZymes and peptidases suggested a shift to pathogenic lifestyle in the evolution of *Sporothrix*. This study is the first time finding the decrease of M20 and S15 subfamily genes in clinical fungi and their potential roles in the evolution of *Sporothrix*. The above findings suggest that *Sporothrix* may have abandoned some useless genes but enriched virulence genes in the evolution.

Genes associated with virulence play important roles in the pathogenicity of fungi. However, the mechanism of variant virulence in *Sporothrix* species remains unknown. In our study, the reduced number of genes associated with the loss of pathogenicity, reduced virulence and unaffected pathogenicity is remarkable in the four main clinical *Sporothrix* species, and it is far more than that associated with the increased pathogenicity and lethal. This finding sheds light to unveil the increased pathogenicity of *Sporothrix* species during their evolution. Furthermore, some proteins encoded by virulence genes have known roles in extracellular vesicle production, dimorphic transition, subversion of host innate immunity, and invasiveness (Rossato et al., 2018; Song et al., 2020). For example, The Cap protein plays an important role in regulating cAMP/protein kinase A (PKA) signaling (Yang et al., 2019). Acyl-CoA functioned in leucine catabolism is required for the vegetative growth, conidiation and full virulence (Li Y. et al., 2019). Hsp70 functioned as a stress-related transcriptional co-activator is required for fungal virulence (Weissman et al., 2020). Deletion of ubiquitin genes results in abnormal morphology, reduced sporulation, and attenuation of virulence (Oh et al., 2012; Chen et al., 2018). SOD contributes to the growth and survival under conditions of oxidative stress (Poli et al., 2004), and the antioxidant function of Cu SOD has been reported to be critical for the pathogenesis of fungi (Narasipura et al., 2003).

The large number of secondary biosynthetic gene clusters in *Sporothrix* suggests their biosynthetic potentials. Further research should provide guidance for the control of harmful secondary metabolites and the develop of beneficial secondary metabolites. Melanin is one of the harmful secondary metabolites prevalent in fungal pathogens, the expression of which is implicated in the pathogenesis (Langfelder et al., 2003). Specifically, melanin is resistant to phagocytosis, free radicals, antifungal drugs, and the host immune system (Al-Laaeiby et al., 2016). Melanin in *Sporothrix* was confirmed by observation *in vitro* and immunofluorescence with murine monoclonal antibodies (Morris-Jones et al., 2003; Godio and Martin, 2009). In this study, we predict that *tIpks* gene cluster is responsible for the biosynthesis of melanin in all *Sporothrix* species. Though contraction of gene clusters appears to be the trend in the evolution of *Sporothrix*, gene cluster associated with melanin is retained. This result contributes to understanding the pathogenic potential of *Sporothrix*. The gene cluster responsible for clavaric acid is also predicted in all *Sporothrix* species. Clavaric acid is a triterpenoid known for its antitumor activity, which is an inhibitor of the human Ras-farnesyl transferase (Li et al., 2018). Future studies on the biosynthesis of clavaric acid in *Sporothrix* by gene manipulation may be useful for the development of antitumor medicine.

## Conclusion

The genomic analyses of the environmental and clinical *Sporothrix* strains might serve as a basis for the understanding their pathogenicity and evolution. Our results suggested that gene contraction was significant in the evolution of pathogenicity. The reduced CAZyme and peptidase genes were usually associated with the decay of plant invasive capabilities in the four main clinical *Sporothrix* species. This study explained a habitat shift in *Sporothrix* species from a saprobic lifestyle in the environment to a parasitic lifestyle in mammals. Furthermore, our analyses showed that the *Sporothrix* species, during their evolution, increased the virulence through reducing genes that were associated with the loss of pathogenicity and the reduced virulence. With respect to the secondary metabolites, we identified two important gene clusters of secondary metabolites, melanin and clavaric acid. We did not find any significant difference in genomes of *S. globosa* stains collected from different regions of China, indicating that regional differences did not lead to significant genetic variations in *S. globosa*. Interactions between microorganism and environment/host might have imposed a strong selection to genomic variations, which drove the evolution of *Sporothrix* species.

## Data Availability Statement

The datasets presented in this study can be found in online repositories. The names of the repository/repositories and accession number(s) can be found in the article/ Supplementary Material.

## Ethics Statement

The studies involving human participants were reviewed and approved by the Committee of Human Rights Related to Research Involving Human Subjects, Chongqing Hospital of Chinese Traditional Medicine. The patients/participants provided their written informed consent to participate in this study.

## Author Contributions

MH and XZ conceived the study, analyzed the data, and wrote the manuscript. MH and ZM performed the genome sequencing, genome assembly, gene prediction, and gene annotation. All authors discussed the results and commented on the manuscript.

## Conflict of Interest

The authors declare that the research was conducted in the absence of any commercial or financial relationships that could be construed as a potential conflict of interest.

## Footnotes

1 http://www.cbcb.umd.edu/software/jellyfish

2 http://bioinf.spbau.ru/spades

3 https://www.kegg.jp/

4 https://antismash.secondarymetabolites.org

5 http://www.cazy.org
